# Supplementary material for: Clinical effects of dexamethasone among patients with sickle cell disease hospitalized with COVID-19: Outcomes from a single academic health system
Source: PLoS One. 2024 Nov 26;19(11):e0313289. doi: 10.1371/journal.pone.0313289 (PMC11594400; doi:10.1371/journal.pone.0313289)
Supplement: Attachment — Submitted filename: Response to Reviewers.docx [file pone.0313289.s001.docx]

**Division of Hospital Medicine**

600 N. Wolfe Street, Meyer 8-134

Baltimore, MD 21287

October 8, 2024

PLOS ONE

Manuscript # PONE-D-24-33591

Dear Editorial Team,

With my co-authors, I am pleased to re-submit with revisions our manuscript titled *Clinical effects of dexamethasone among patients with sickle cell disease hospitalized with COVID-19: Outcomes from a single academic health system* with our response to reviewers’ comments.

Below are our point-by-point responses. We have bolded the editorial requirements and reviewers’ comments and placed our responses underneath.

**Journal Requirements:**

**1. Please ensure that your manuscript meets PLOS ONE's style requirements, including those for file naming. The PLOS ONE style templates can be found at**

**https://journals.plos.org/plosone/s/file?id=wjVg/PLOSOne_formatting_sample_main_body.pdf and**

**https://journals.plos.org/plosone/s/file?id=ba62/PLOSOne_formatting_sample_title_authors_affiliations.pdf**

The manuscript has been edited to reflect the style requirements

**2. Thank you for stating the following in the Competing Interests section:**

**"I have read the journal's policy and the authors of this manuscript have the following competing interests: KAG receives royalties from UpToDate, non-paid position at Pfizer, and personal consulting from Spark HealthCare, Premier HealthCare, Harrison Consulting and MedEd Learning. WMG reports receiving honorarium from DKBmed; serving as a scientific advisor to Gilead Sciences, Inc; and owning stock in Abbott Laboratories, Danaher, Eli Lilly and Company, Iqvia, Johnson & Johnson, Stryker, UnitedHealth Group, and AstraZeneca Pharmaceuticals. SML reports consulting with bluebird bio, Agios Pharmaceuticals, Novo Nordisk, Pfizer, Merck, GlycoMimetics; prior research support from CSL Behring, Novartis, Takeda Pharmaceuticals; ongoing research support from Patient-Centered Outcomes Research Institute (PCORI), Health Resources and Services Administration and stock ownership in Pfizer, Teva Pharmaceuticals."**

**Please confirm that this does not alter your adherence to all PLOS ONE policies on sharing data and materials, by including the following statement: "This does not alter our adherence to PLOS ONE policies on sharing data and materials.” (as detailed online in our guide for authors http://journals.plos.org/plosone/s/competing-interests). If there are restrictions on sharing of data and/or materials, please state these. Please note that we cannot proceed with consideration of your article until this information has been declared.**

**Please include your updated Competing Interests statement in your cover letter; we will change the online submission form on your behalf.**

The statement has been added to a revised cover letter.

**3. We note that you have indicated that there are restrictions to data sharing for this study. PLOS only allows data to be available upon request if there are legal or ethical restrictions on sharing data publicly. For more information on unacceptable data access restrictions, please see http://journals.plos.org/plosone/s/data-availability#loc-unacceptable-data-access-restrictions.**

**Before we proceed with your manuscript, please address the following prompts:**

**a) If there are ethical or legal restrictions on sharing a de-identified data set, please explain them in detail (e.g., data contain potentially identifying or sensitive patient information, data are owned by a third-party organization, etc.) and who has imposed them (e.g., a Research Ethics Committee or Institutional Review Board, etc.). Please also provide contact information for a data access committee, ethics committee, or other institutional body to which data requests may be sent.**

The data set for this study includes protected health information and was obtained under a waiver of consent. The study was authorized by the Johns Hopkins Medicine Institutional Review Board and use of the data set is controlled by legal restrictions. Disclosure of the de-identified dataset to a third party is not permitted under the approved research protocol. Requests for data access can be submitted to the Institutional Review Board at jhmeirb@jhmi.edu.

**b) If there are no restrictions, please upload the minimal anonymized data set necessary to replicate your study findings to a stable, public repository and provide us with the relevant URLs, DOIs, or accession numbers. For a list of recommended repositories, please see https://journals.plos.org/plosone/s/recommended-repositories. You also have the option of uploading the data as Supporting Information files, but we would recommend depositing data directly to a data repository if possible.**

**We will update your Data Availability statement on your behalf to reflect the information you provide.**

**4. Your ethics statement should only appear in the Methods section of your manuscript. If your ethics statement is written in any section besides the Methods, please delete it from any other section.**

The manuscript has been edited to include the ethics statement in the methods section only.

**5. We note you have included a table to which you do not refer in the text of your manuscript. Please ensure that you refer to Table 3 and 4 in your text; if accepted, production will need this reference to link the reader to the Table.**

Reference to Table 3 and Table 4 has been added to the manuscript.

**Reviewer Comments:**

**Reviewer #1: The authors present a thorough retrospective cohort study describing the clinical outcomes of patients with sickle cell disease admitted with COVID, comparing those treated or not treated with dexamethasone.

As they state in the introduction, there is a question among hematologists about the risk of rebound vaso-occlusive episodes of pain after steroid treatment in this population. I do not see results that report on the rates of rebound VOE in this cohort. The authors do state that the rate of readmission among those treated with dex is lower, but it appears that this is all-cause readmission. Please add the rates of VOE after dexamethasone between the groups**

Thank you for highlighting this omission. A sentence summarizing these findings has been added to the manuscript (Results, Paragraph 5). This information is also listed in Table 3.

**The high rate of VTE is an interesting finding, however, it is at high-risk of confounding by indication, as the authors briefly mention in the discussion. More severe COVID, ICU stay, and prolonged hospitalization are all risk factors for VTE, and more severe COVID could also be the reason patients were treated with dexamethasone. It is a strength that the authors include severity score on admission, but patients with COVID can devolve dramatically during hospitalization. The fact that the dexamethasone-treated patients also were more likely to need a transfusion also supports that they were more severe. The authors should elaborate further on this important consideration/limitation in the discussion**

The following statement has been added to the discussion to reflect this point: **“**Additionally, while we have attempted to control for severity of illness, there may be residual confounding by indication, as evidenced by higher rates of transfusion in this cohort that contribute to the higher rate of VTE in the dexamethasone treated group” (Discussion, Paragraph 4).

**Reviewer #2: Thank you for allowing me to review the paper Clinical effects of dexamethasone among patients with sickle cell disease hospitalized with COVIDS-19. Outcomes from a single academic health system." The paper is well-written.

In the results section. the authors note that "Younger patients were more likely to be treated with dexamethasone than older patients. Can the authors proffer a reason for this observation?**

Thank you for drawing attention to this finding. In our study, younger patients had more severe COVID-19 as measured by respiratory requirements at admission and overall WHO severity score. Commentary has been added to the discussion (Discussion, Paragraph 4).

**End of Comments.**

We appreciate the comments of the editorial team and reviewers which have strengthened the manuscript. We look forward to your review of our response. Please do not hesitate to contact me with any questions.

Sincerely,

William Garneau MD MPH

Assistant Professor of Medicine

Division of Hospital Medicine

Johns Hopkins University School of Medicine
